# Therapeutic Use of *Parerythrobacter* sp. M20A3S10, a Marine Bacterium, Targeting Influenza Viruses and Flaviviruses

**DOI:** 10.3390/ani15142125

**Published:** 2025-07-18

**Authors:** Kyeong-Seo Moon, Ji-Young Chung, Hyeon Jeong Moon, Gun Lee, Chung-Do Lee, Su-Bin Jung, Hyo-Jin Kim, Jun-Gyu Park, Yeong-Bin Baek, Sang-Ik Park

**Affiliations:** 1 Department of Veterinary Pathology, College of Veterinary Medicine, Chonnam National University, Gwangju 61186, Republic of Korea; ksmoon0409@gmail.com (K.-S.M.); cndehsla@gmail.com (C.-D.L.); jsbin810@gmail.com (S.-B.J.); gywlsl0420@gmail.com (H.-J.K.); 2 Jeollanamdo Veterinary Service Laboratory, Gangjin 59213, Republic of Korea; jjy1210@korea.kr; 3 Department of Veterinary Pathology, College of Veterinary Medicine and BK21 FOUR Program, Chonnam National University, Gwangju 61186, Republic of Korea; dals404@naver.com (H.J.M.); udlrjs77@naver.com (G.L.); 4 Department of Veterinary Zoonotic Diseases, College of Veterinary Medicine, Chonnam National University, Gwangju 61186, Republic of Korea; kingsalt@jnu.ac.kr

**Keywords:** marine bacterium, influenza virus, Zika virus, dengue virus, apoptosis, broad antiviral activity

## Abstract

Emerging RNA viruses such as influenza A virus (IAV), Zika virus (ZIKV), and dengue virus (DENV) continue to pose major challenges to animal and public health due to their high mutation rates, wide host ranges, and immune evasion strategies. In this study, we evaluated the in vitro antiviral activity of a marine bacterial extract derived from *Parerythrobacter* sp. M20A3S10 against IAV (H1N1; H3N2), influenza B virus (IBV), ZIKV, and DENV2. The extract demonstrated broad-spectrum antiviral effects with favorable selectivity indices across multiple host-derived epithelial cell lines. Notably, post-infection treatment significantly suppressed viral replication, suggesting a host-modulating or replication-inhibiting mechanism. While the extract’s active components have yet to be identified, bacteria from the *Erythrobacteraceae* family are known producers of bioactive metabolites with potential antiviral properties. These findings provide preliminary insight into the potential of marine-derived bacterial compounds in veterinary antiviral development and highlight the need for further characterization and in vivo validation. This work contributes to the understanding of virus–host interactions and the exploration of novel therapeutic strategies targeting the pathogenesis and immune modulation of veterinary RNA viruses.

## Simple Summary

Viruses that affect both animals and humans, such as influenza, Zika, and dengue, are spreading more easily across the world due to global travel, climate change, and close contact between people, animals, and insects. These viruses are difficult to control because they can change quickly and escape the body’s natural defenses. In this study, we looked for new ways to fight these viruses using substances made by bacteria found in the ocean. We tested a natural extract from one marine bacterium and found that it could reduce the ability of several viruses to grow in infected cells. This included viruses that commonly affect animals and that can also spread to people. The extract worked after the cells were already infected, showing promise as a possible treatment option. Although we do not yet know the exact ingredient in the extract responsible for this effect, our results suggest that marine bacteria could be a valuable new source of antiviral compounds. This discovery is significant as it may facilitate the development of improved therapeutics for animals and help reduce the risk of zoonotic viral transmission.

## 1. Introduction

Emerging and re-emerging RNA viruses continue to pose substantial threats to both human and animal populations, often originating in animal reservoirs and exhibiting zoonotic potential. Among these, influenza A virus (IAV) remains a prototypical example of a veterinary virus capable of cross-species transmission with pandemic consequences. Its segmented RNA genome, coupled with the high mutation rate and ability to undergo antigenic drift and shift, enables rapid adaptation to new hosts, including humans, pigs, birds, and other mammals [1,2]. These properties not only complicate vaccination and antiviral strategies but also underscore the need for coordinated surveillance systems to monitor circulating strains [3]. The continuing emergence of highly pathogenic avian influenza (HPAI) strains in domestic and wild animals reflects this dynamic evolution and highlights the relevance of veterinary virology in global pandemic preparedness.

Similarly, flaviviruses such as Zika virus (ZIKV) and dengue virus (DENV) represent serious public and veterinary health challenges. These arthropod-borne viruses are widely distributed in tropical and subtropical regions and are known to cause significant morbidity and mortality across various species. The 2015–2016 ZIKV outbreak in the Americas, associated with congenital brain malformations, as well as the ongoing burden of DENV with an estimated 390 million annual infections, exemplify their capacity for rapid geographic expansion and severe clinical outcomes [4,5]. Importantly, the spread of these flaviviruses is facilitated by global environmental changes, vector expansion, and increased human–animal interface, necessitating integrative One Health strategies to understand and control their epidemiology.

A common feature of these RNA viruses is their interaction with host cell death pathways, particularly apoptosis and necroptosis. While apoptosis traditionally acts as a host defense mechanism to limit viral replication by eliminating infected cells, certain viruses manipulate this pathway to their advantage [6]. Recent studies have shown that viruses such as IAV, ZIKV, and DENV induce necroptotic signaling, potentially contributing to tissue damage and immune modulation [7,8]. Conversely, apoptotic processes—when well-regulated—can facilitate containment of infection through the formation of apoptotic bodies that encapsulate viral particles, preventing their release and promoting clearance by phagocytes [6,9]. Thus, understanding how veterinary viruses interface with host immunological responses, including programmed cell death, is critical to elucidating their pathogenesis.

In response to these complex host–virus interactions and the limitations of current antiviral therapies, there has been increasing interest in the discovery of broad-spectrum antiviral compounds from natural sources. Marine microorganisms, particularly bacteria of the family *Erythrobacteraceae*, have emerged as promising candidates. These bacteria produce carotenoids with demonstrated antiviral and antibacterial properties. Compounds from *Qipengyuania pacifica* and related genera have been shown to inhibit viral replication and modulate host inflammatory responses [10,11,12]. Notably, these natural products often exert antiviral effects through mechanisms such as interference with viral genome transcription or modulation of host immune pathways.

Marine bacteria are also advantageous due to their rapid growth in low-cost conditions and ease of large-scale fermentation, making them ideal candidates for sustainable antiviral drug development [13,14]. Moreover, carotenoids and other bioactive metabolites derived from marine pigmented bacteria have shown antiviral activity against a wide spectrum of viruses, including herpes simplex virus, IAV, and flaviviruses such as ZIKV and DENV [15,16,17]. Despite these promising findings, the antiviral potential of marine bacteria remains underexplored, particularly in the veterinary virology context.

In this context, our study evaluated the antiviral activity of marine microbial extracts against several enveloped RNA viruses of veterinary importance, including IAV (H1N1, H3N2), influenza B virus (IBV), ZIKV, and DENV2. Among the screened candidates, an extract derived from *Parerythrobacter* sp. M20A3S10 exhibited significant in vitro antiviral effects with favorable selectivity indices. This strain, belonging to the *Erythrobacteraceae* family, is expected to produce carotenoid compounds responsible for its antiviral activity. These findings suggest a novel therapeutic potential of marine-derived metabolites, not only as direct-acting antivirals but also as modulators of host immunity and viral pathogenesis.

To date, no studies have reported antiviral activity in *Parerythrobacter* spp., underscoring the novelty of this study in proposing its application in antiviral research. This work contributes to the growing field at the intersection of viral pathogenesis, immunology, and veterinary epidemiology, offering new insights into host–virus interactions and highlighting marine microbes as promising candidates for next-generation antiviral development.

## 2. Materials and Methods

### 2.1. Bacterial Isolation and Culture Conditions

Several bacterial strains, including *Parasphingopyxis*, *Roseibium*, *Parerythrobacter*, *Neorhizobium*, and *Qipengyuania* species, were collected from seawater in Seosan (Chungnam Province, Republic of Korea) on 16 March 2020. The bacteria from the water sample were cultured as previously described [17,18]. The purified strains were routinely cultured on marine agar 2216 (Difco, Franklin Lakes, NJ, USA) at 25 °C and preserved with 20% (*v*/*v*) glycerol at −80 °C. The bacterial isolates were deposited under the numbers MI00005948, MI00006287, MI00006290, MI00006301, MI00007026, and MI00007049, respectively, at the Microbial Marine BioBank (MMBB) of the National Marine Biodiversity Institute of Korea (MABIK).

### 2.2. Phylogeny of 16S rRNA Gene Sequences

**Figure 1:**
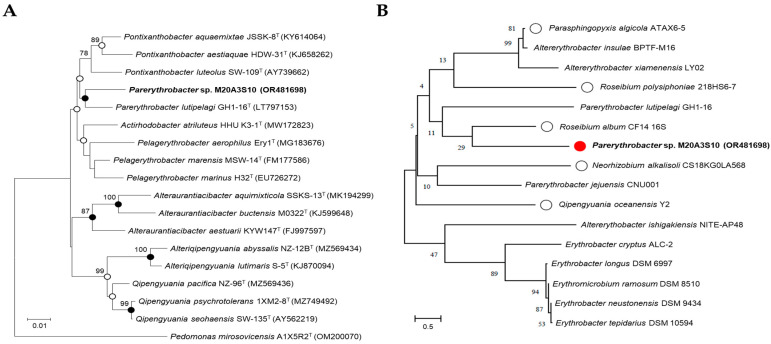
Phylogenetic analysis of marine bacteria either in use or for comparison with isolated strains. (**A**) A neighbor-joining tree based on 16S rRNA gene, showing the phylogenetic relationships of strain M20A3S10 (in bold type) and closely related taxa with validly published names. GenBank accession numbers are given in parentheses. Bootstrap values above 70% are shown on nodes in percentages of 1000 replicates. Closed and open circles indicate nodes that were supported by all three methods (neighbor-joining, maximum likelihood, and maximum parsimony) or by two methods, respectively. Bar, 0.01 changes per nucleotide position. (**B**) Marine bacteria whose extracts were subjected to preliminary antiviral screening. White circles indicate bacterial strains whose extracts showed significant antiviral activity in the initial screening. The red circle represents the bacterial strain whose extract exhibited the most potent antiviral efficacy Bar, 0.5 changes per nucleotide position.

Genomic DNA was extracted using the Exgene DNA extraction kit (GeneAll, Seoul, Republic of Korea), following the manufacturer’s protocol. The 16S rRNA gene was amplified using bacteria-specific universal primers 27F and 1492R, and sequenced using an automated sequencer (ABI 3730XL, Applied Biosystems, Foster City, CA, USA) at Macrogen Co., Ltd. (Seoul, Republic of Korea) as previously described [17]. The obtained sequences were assembled with Geneious v9.0.5 to yield a nearly full-length 16S rRNA gene sequence. For taxonomic identification, the 16S rRNA gene sequence (1494 bp) of *Parerythrobacter* sp. M20A3S10 was analyzed using the EzBioCloud server (https://www.ezbiocloud.net/identify, accessed on 30 March 2022) and compared with validated type strains (Figure 1A). The sequence has been deposited in GenBank under the accession number OR481698.

In addition to this in silico comparison, phylogenetic analysis was also performed among M20A3S10 and other bacterial isolates obtained from the same seawater sample (Figure 1B), which served as local reference strains. Phylogenetic trees were constructed based on 1419 unambiguously aligned sites using the neighbor-joining (NJ), maximum-likelihood (ML), and maximum-parsimony (MP) methods in MEGA X version 11.0 [19,20]. Tree robustness was assessed with 1000 bootstrap replicates across all three algorithms.

### 2.3. Preparation of the Bacterial Extracts

Bacterial extracts were prepared using a modified method as previously described [17,18]. Five bacterial strains—*Parasphingopyxis*, *Roseibium*, *Parerythrobacter*, *Neorhizobium*, and *Qipengyuania* spp.—were initially cultured in 2.5 L Erlenmeyer flasks containing 1 L of marine broth (total volume: 5 L) under continuous illumination (60 µmol m^−2^ s^−1^ LED light) at 25 °C. The resulting seed cultures were subsequently transferred to a 20 L panel- or column-type photobioreactor and incubated at a starting concentration of 10^4^ CFU mL^−1^ under the same conditions for 10–20 days with shaking at 150 rpm. Following cultivation, the culture broth was extracted twice with an equal volume of ethyl acetate (EtOAc). The EtOAc-soluble fractions were pooled and concentrated using a vacuum evaporator. Each bacterial strain yielded approximately 150 mg of crude extract, which was dissolved in dimethyl sulfoxide (DMSO) for subsequent antiviral assays.

### 2.4. Cells and Viruses

Madin–Darby canine kidney epithelial cells (MDCK; ATCC CCL-34), African green monkey kidney epithelial cells (Vero E6; ATCC CRL-1586), and human lung carcinoma epithelial cells (A549; ATCC CCL-185) were obtained from the American Type Culture Collection (ATCC, Manassas, VA, USA). All cell lines were maintained in Dulbecco’s Modified Eagle’s Medium (DMEM) supplemented with 10% fetal bovine serum (FBS), 100 U/mL penicillin, and 100 μg/mL streptomycin.

Two strains of IAV and one strain of IBV from ATCC were used: influenza virus A/Puerto Rico/8/34 (H1N1) (A/PR8), A/Wisconsin/15/2009 (H3N2) (A/Wisconsin) strain, and B/Florida/78/2015 Victoria lineage (B/Florida). Both types of flavivirus were provided from the National Culture Collection for Pathogens (Cheongju, Republic of Korea): Dengue virus type 2 (DENV2) isolated from serum samples from Korean patients traveling to Singapore, India, and Thailand in 2015 and Zika virus (ZIKV) strains of Asian/American lineage, PRVABC59.

MDCK cells are widely used for influenza virus propagation due to their high permissiveness, while Vero E6 cells are suitable for the replication of flaviviruses such as ZIKV and DENV. So, the IAV and IBV strains were propagated in MDCK cells supplemented with 1 μg/mL N-tosyl-L-phenylalanine chloromethyl ketone (TPCK)-treated trypsin (Sigma-Aldrich, St. Louis, MO, USA), while ZIKV and DENV were propagated in Vero E6 cells. Viral titers were quantified using a cell culture-based immunofluorescence assay.

### 2.5. MTT Assay

A confluent monolayer of MDCK and Vero E6 cells was prepared in a 96-well plate at a healthy density of 5.0 × 10^4^ cells. The extracts were prepared using a continuous dilution method with an adjusted dilution factor, resulting in the following concentrations: 1000, 500, 250, 100, 50, 25, 10, 5, 2, 1, 0.5, 0.1, 0.01 µg/mL. Cell viability was calculated as a percentage in comparison with DMSO-treated cells using GraphPad Prism software v9.5.1 (GraphPad Software, San Diego, CA, USA) as described previously [21].

### 2.6. Preliminary Antiviral Screening

To evaluate antiviral activity, a screening assay was performed using a modified method as previously described [22,23]. Permissive cell lines were pretreated with serially diluted bacterial extracts (100, 50, 25, 10, 5, 2, 1, 0.5, 0.1, and 0.01 µg/mL) for 1 h, followed by infection with A/PR8 (H1N1), ZIKV, or DENV2 at a multiplicity of infection (MOI) of 0.1. Viral adsorption was carried out at room temperature for 1 h in a medium supplemented with 1% penicillin–streptomycin and 1 µg/mL TPCK-treated trypsin. After removing the inoculum and washing with Dulbecco’s phosphate-buffered saline (DPBS), the extracts were reapplied and cells were incubated for an additional 48 h. Cell viability was assessed using the MTT assay according to the manufacturer’s instructions [24], and nonlinear regression analysis was conducted to determine the median inhibitory concentration (IC_50_).

### 2.7. Time-of-Addition Antiviral Experiment

Time-of-addition antiviral assays were performed to evaluate the stage at which the *Parerythrobacter* sp. M20A3S10 extract exerts its antiviral effect. Treatments were administered at three different time points: prior to infection (pre-treatment), concurrently with infection (co-treatment), or after infection (post-treatment), following previously described protocols [17,18].

For pre-treatment, cells were incubated with the extract for 1 h, washed with DMEM, and subsequently infected with IAV, ZIKV, or DENV2 at an MOI of 0.1. After 1 h of viral adsorption, cells were washed and replaced with DMEM containing 1 µg/mL TPCK-treated trypsin for IAV, or DMEM alone for ZIKV and DENV2.

For co-treatment, cells were exposed to the virus and extract simultaneously for 1 h at 37 °C, followed by washing and medium replacement as described above.

For post-treatment, cells were first infected with virus for 1 h, washed, and then treated with various concentrations of the extract in DMEM with or without TPCK-treated trypsin, depending on the virus.

All treatments were performed in triplicate using 96-well plates. At 48 h post-infection, culture supernatants were harvested for the quantification of viral RNA levels and progeny virus titers.

### 2.8. RT-qPCR

The quantitative RT-qPCR method was used to measure the virus in infected cells, and cell supernatants were analyzed at 48 h after treatment. Virus RNA was extracted using the QIAamp Viral RNA Mini kit (QIAGEN, Hilden, DE, USA) per the manufacturer’s guidelines [8]. PCR was performed with SensiFAST™ SYBR^®^ Lo-ROX One-Step Kit (Bioline Meridian BioScience, Cincinnati, OH, USA) as previously described [17,18], using the detecting primers as follows: forward (5′-GGCCCTTCAGT TGTTCATC-3′) and reverse primers (5′-GCAGACTTCAGGAATGTG-3′) against IAV PB1, forward (5′-GGTCATGATACTGCTGATTGC-3′) and reverse primers (5′-CCACTAACGTTCTTTTGCA GAC-3′) against ZIKV NS5, forward (5′-AGTTGTT AGTCTRYGTGGACCGAC-3′) and reverse primers (5′-TTGCACCAACAGTCAATGTCTTCAG GTTC-3′) against DENV prM-. The primers were designed in-house using NCBI Primer-BLAST (https://www.ncbi.nlm.nih.gov/tools/primer-blast, accessed on 15 July 2025) based on conserved regions of the viral genomes.

### 2.9. Virus Titration

To assess progeny virus production, a 50% tissue culture infective dose (TCID_50_) assay was performed. IAV, ZIKV, and DENV titers were determined using their respective permissive cell lines. Briefly, ten-fold serial dilutions of each virus stock were prepared in DMEM, and 100 µL of each dilution was inoculated onto monolayers of permissive cells in 96-well plates. For IAV, the medium contained 1 µg/mL TPCK-treated trypsin; for ZIKV and DENV2, DMEM without trypsin was used. Plates were incubated at 37 °C in a 5% CO_2_ atmosphere, and viral cytopathic effects were evaluated at 4 days post-infection. TCID_50_/mL values were calculated using the Reed and Muench method [8].

### 2.10. Attachment and Penetration Assays

The attachment and penetration assays were performed as described previously [25]. For the attachment assay, confluent MDCK cells in a 24-well plate were treated with extracts (100 µg/mL) at 4 °C before infection. After 30 min, the inoculum was washed thoroughly with cold DPBS. Then, the A/PR8 strain (1 MOI) was absorbed for 1 h on ice. After thorough washing with cold DPBS, the cells were incubated for 20 h at 37 °C in a 5% CO_2_ atmosphere. MDCK cells in a 24-well plate received A/PR8 strain (1 MOI) for 1 h on ice for penetration assay. After thorough washing, the cells were treated with extracts for 10 min and incubated for 20 h at 37 °C in a 5% CO_2_ atmosphere.

### 2.11. Cell Culture Immunofluorescence Assay

MDCK cells were prepared in an 8-well chamber to check viral protein synthesis and infected with the A/PR8 strain (0.1 MOI). Vero E6 cells were also prepared and infected with ZIKV and DENV2 (0.1 MOI) as described elsewhere [8]. After fixation with 4% PFA for 10 min at RT, the chamber was washed and blocked with 5% Bovine Serum Albumin (BSA) at RT for 1 h to reduce non-specific reaction.

Subsequently, cells were incubated overnight at 4 °C with primary antibodies targeting either the influenza A virus M2 protein (ab5416, Abcam, Cambridge, UK) or the flavivirus group envelope protein (clone 4G2, Native Antigen Company, Oxfordshire, UK). The next day, after washing, a fluorescently labeled secondary antibody (goat anti-mouse IgG conjugated with Alexa Fluor (AF) 488; A-11001, Thermo Scientific, Waltham, MA, USA) was applied and incubated for 1 h at RT. Fluorescence images were captured using a fluorescence microscope to assess the presence and distribution of viral proteins.

### 2.12. Western Blotting

To evaluate the expression levels of viral proteins in cultured cells, Western blot analysis was performed as previously described [8]. Briefly, cells were lysed using RIPA buffer containing 10 mM Tris-HCl (pH 7.4), 100 mM NaCl, 1 mM EDTA, 1 mM EGTA, 1 mM NaF, 20 mM Na_2_P_2_O_7_, 2 mM Na_3_VO_4_, 1% Triton X-100, 10% glycerol, 0.1% SDS, and 0.5% deoxycholate (Invitrogen, Waltham, MA, USA) for 10 min on ice. The lysates were centrifuged at 12,000× *g* for 10 min at 4 °C, and the supernatant was collected. Total protein concentration was determined using a BCA protein assay kit (Thermo Scientific).

Equal amounts of protein were subjected to SDS-PAGE and transferred onto nitrocellulose membranes (GE Healthcare Life Sciences, Lafayette, CO, USA). The membranes were blocked for 1 h at room temperature in Tris-buffered saline containing 5% skim milk and 0.1% Tween-20, then incubated overnight at 4 °C with a primary antibody against IAV NP (ab128193, Abcam, Cambridge, UK) or β-actin (sc-47778, Santa Cruz Biotechnology, Dallas, TX, USA). After washing, membranes were incubated for 1 h at room temperature with HRP-conjugated goat anti-mouse IgG secondary antibody (SC-2005, Santa Cruz Biotechnology, Dallas, TX, USA). Protein bands were visualized using enhanced chemiluminescence (ECL) substrate (Dogen, Seoul, Republic of Korea) and imaged with a Davinch-K imaging system (Youngwha Scientific Co., Ltd., Seoul, Republic of Korea). Band intensities were quantified and normalized to β-actin as an internal control, and the results were expressed as relative fold changes.

### 2.13. Flow Cytometry

The present study utilized flow cytometry to assess the induction of viral protein and apoptosis. The methodology adopted was in accordance with previously established protocols [17]. Briefly, MDCK cells were inoculated with A/PR8 strain (0.1 MOI), and Vero E6 cells were inoculated with ZIKV (0.1 MOI) or DENV2 (0.1 MOI). Primary antibodies against IAV NP (ab128193, Abcam, Cambridge, UK) and flavivirus envelope protein (4G2, Native Antigen Company, Oxford, UK) and secondary antibodies conjugated with goat-anti mouse AF647 (A32728, Thermo Scientific) were applied to evaluate viral protein. For apoptosis detection, a TUNEL assay was performed using the In Situ Cell Death Detection Kit (11684795910, Roche, Basel, Switzerland) according to the manufacturer’s instructions. Flow cytometry was performed using the AttuneTM NxT flow cytometer (Thermo Fisher Scientific), and the AttuneTM NxT software v3.1.2 was used to digitize the data from each sample.

### 2.14. Statistical Analysis

Statistical analysis was carried out using GraphPad Prism software, with one-way ANOVA being employed. The data was expressed as the mean ± standard deviation (SD) of at least three independent experiments. Statistical significance was considered at the following levels: *, *p* < 0.05; **, *p* < 0.01; ***, *p* < 0.001; ****, *p* < 0.0001. The selective index (SI) was calculated using sigmoidal dose–response curves, with the following equation: SI = mean CC_50_/mean IC_50_. The ratio of CC_50_ to IC_50_, i.e., CC_50_/IC_50_, was employed to calculate SI.

## 3. Results

### 3.1. Phylogenetic Analyses and Antiviral Screening of Marine Bacteria

The comparative analysis of the 16S rRNA gene sequences showed that *Parerythrobacter* sp. M20A3S10 (accession number: OR481698) was most closely related to *Parerythrobacter lutipelagi* GH1-16T (accession number: LT797153) with a similarity of 98.56%. The phylogenetic analysis showed that the strain M20A3S10 formed a monophyletic clade with other members of the genus *Parerythrobacter* (Figure 1).

We further investigated the 16S rRNA gene sequences using marine bacteria which were sampled in the same batch and were genetically close to *Parerythrobacter* sp. M20A3S10 (Figure 1). For the antiviral assay, MDCK cells—known for their high permissiveness to IAV and IBV—were used. The choice of epithelial cell lines was based on their relevance to the entry and replication mechanisms of each virus, particularly reflecting the epithelial tropism characteristic of influenza viruses. The A/PR8 strain was selected for initial screening due to its well-established use in both in vitro and in vivo studies, and its efficient replication across a range of mammalian cell types [26].

**Figure 2:**
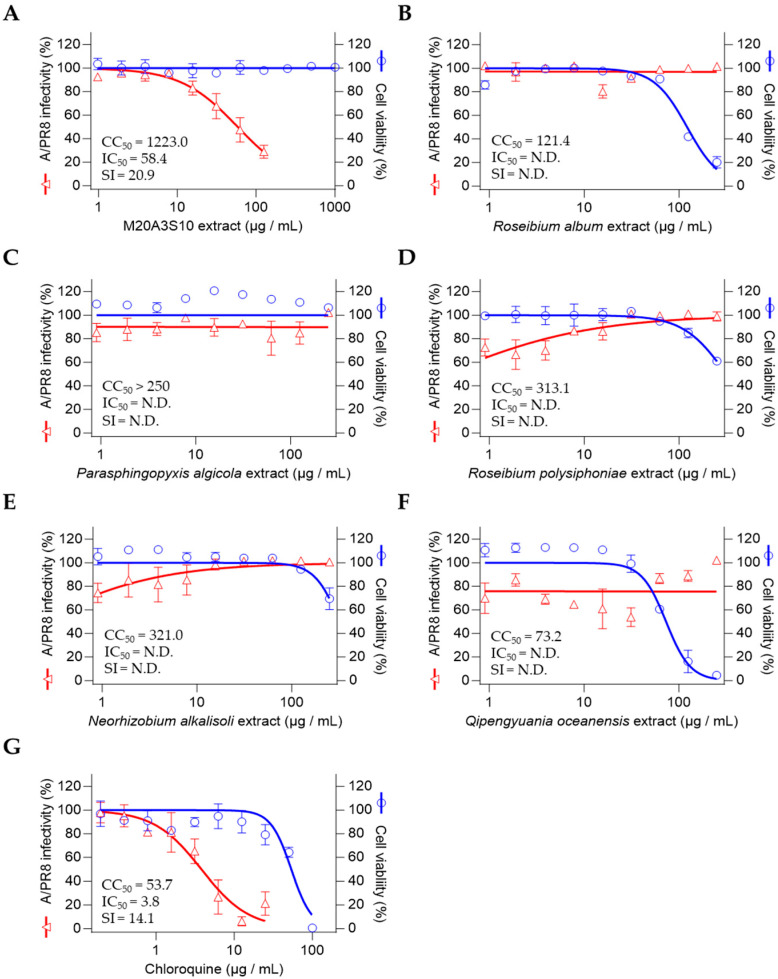
Marine bacterial extracts subjected to preliminary antiviral screening under both pre-treatment and post-treatment conditions: (**A**) *Parerythrobacter* sp. M20A3S10, (**B**) *Roseibium album*, (**C**) *Parasphingopyxis algicola*, (**D**) *Roseibium polysiphoniae*, (**E**) *Neorhizobium alkalisoli*, (**F**) *Qipengyuania oceanensis*, (**G**) Chloroquine. N.D., not determined.

So, antiviral screening was conducted on strain M20A3S10 and five other selected bacteria through the determination of the IC_50_ of the extracts and was measured by quantitative cytopathic effect (CPE) reduction in A/PR8 strain-infected cells (0.1 MOI) (Figure 2). As a result, the extract of *Parerythrobacter* sp. M20A3S10 (M20A3S10 extract) showed the most significant antiviral activity against A/PR8 infection (CC_50_ = 1223 µg/mL, IC50 = 58.4 µg/mL, SI = 20.9), which is greater than chloroquine (CC_50_ = 53.7 µg/mL, IC50 = 3.8 µg/mL, SI = 14.1), an FDA-approved antiviral drug [27].

### 3.2. Evaluation by Pre- or Co-Treatment of M20A3S10 Extract

To understand the antiviral mechanism of the M20A3S10 extract, MDCK cells were treated with the extract before (pre-treatment) or with viral infection (co-treatment) (Figure S1). Antiviral activity against the A/PR8 strain was measured by the inhibition effect on viral CPE using the MTT assay. However, neither treatment detected antiviral effects at any concentrations. Accordingly, viral genome copies in the infected cells acquired at 48 h post-infection were not attenuated by the treatments.

### 3.3. Antiviral Evaluation by Post-Treatment of M20A3S10 Extract

**Figure 3:**
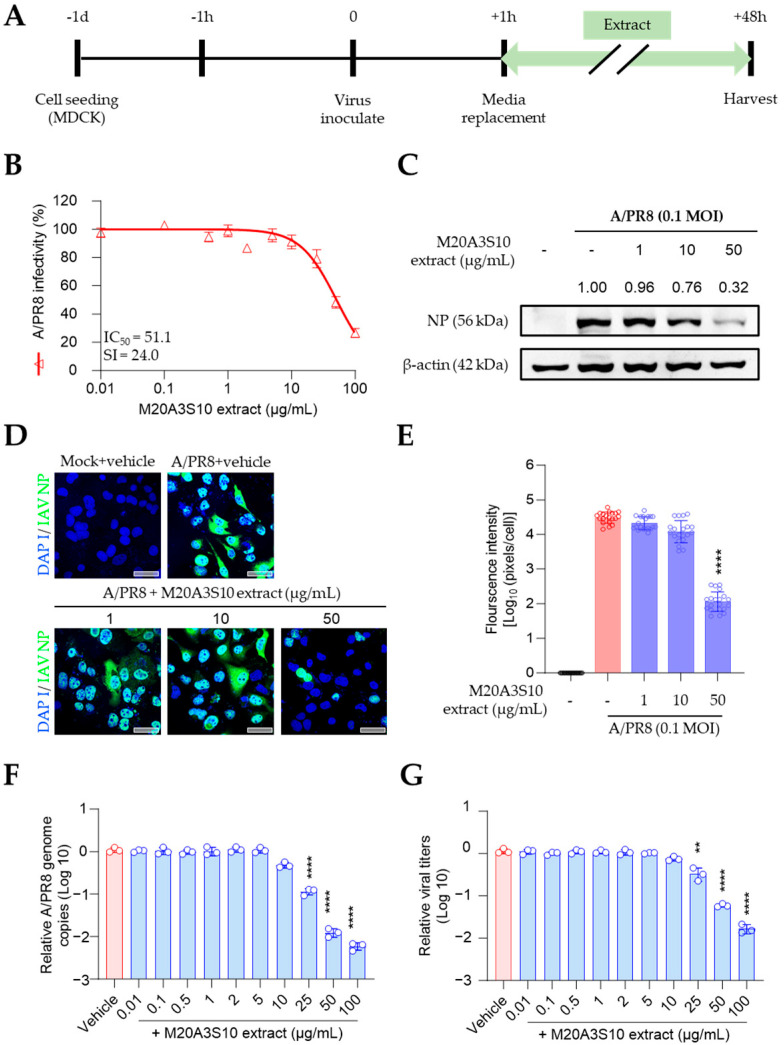
Antiviral effect of M20A3S10 extract by post-treatment. (**A**) Schematic diagram of virus inoculation and extract treatment. (**B**) IC_50_ and SI of M20A3S10 extract measured by CPE-inhibition assay. (**C**) The expression level of NP of MDCK cells infected with or without A/PR8 strain and treated with or without M20A3S10 extract. (**D**) Representative IFA images of virus replication under the vehicle or extract treatment using an anti-IAV NP antibody. Cells were stained with DAPI. Bar = 20 µm. (**E**) The quantification of viral protein was produced by mathematical calculation of positive signals per cell in the IFA Image. (**F**) Viral genome copies detected by RT-qPCR. (**G**) Progeny virus production was measured by TCID50. All data in the graphs are presented as arithmetic means ± S.D. from three independent experiments. **, *p* < 0.01; ****, *p* < 0.0001.

Therefore, the antiviral mechanism was further studied using post-treatment of the extract (Figure 3A). As shown in Figure 2, influenza virus-induced CPE was dramatically decreased by the M20A3S10 extract with no observed cytotoxicity, exhibiting a great therapeutic index (CC_50_ = 1223 µg/mL, IC_50_ = 51.1 µg/mL, SI = 24.0) (Figure 3B). In addition, IFA and Western blot revealed that the treatment suppressed virus protein synthesis in a dose-dependent manner (Figure 3C–E). According to RT-qPCR and TCID50, a gradual antiviral activity was found toward higher concentrations, resulting in significant reductions in viral genome synthesis as well as infectious virus particles (Figure 3F,G).

### 3.4. Apoptosis-Mediated Antiviral Response Through M20A3S10 Extract

Further antiviral mechanism was investigated using attachment and penetration assays (Figure S2). MDCK cells pre-treated with M20A3S10 extract, followed by A/PR8 infection, did not exhibit any reduction in viral genome copies, and while viral inoculum pre-incubated with antibody against IAV HA significantly suppressed genome synthesis (Figure S2A). Interestingly, according to the penetration assay, virus genomes were significantly reduced by post-treatment of the extract after viral binding to MDCK cells, which was cross-checked by chloroquine treatment (Figure S2B). However, this antiviral activity is expected to continue only during early infection. It is not enough to suppress persistent infection, given that the pre-and co-treatments are not enough to curb viral infection during a long incubation period, such as 48 h of infection. In other words, the significant antiviral activity shown in Figure 3 was highly linked to the late stage of viral replication or post-entry stages.

**Figure 4:**
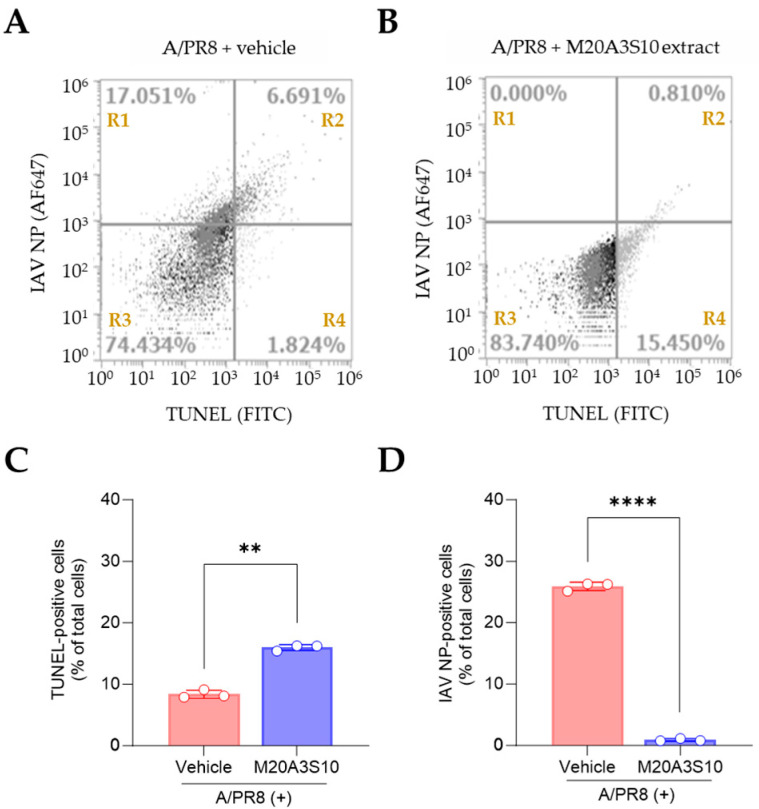
Evaluation of apoptotic response by M20A3S10 extract using flow cytometry. Apoptosis was assessed using the TUNEL assay, and viral protein expression was detected with an antibody against IAV NP. (**A**,**B**) Flow cytometry plots: The x-axis represents the fluorescence intensity of the FITC-conjugated TUNEL signal (Ex: 485 nm, Em: 528 nm), and the y-axis represents the fluorescence intensity of the AF647-conjugated anti-NP antibody (Ex: 650 nm, Em: 665 nm). (**A**) A/PR8-infected (0.1 MOI), vehicle-treated group. (**B**) A/PR8-infected (0.1 MOI), M20A3S10 extract-treated group. (**C**,**D**) Quantification of TUNEL-positive cells (**C**) and IAV NP-positive cells (**D**) in the vehicle- and extract-treated groups. **, *p* < 0.01; ****, *p* < 0.0001. R1: NP^+^/TUNEL^−^ cells; R2: NP^+^/TUNEL^+^ cells; R3: NP^−^/TUNEL^−^ cells; R4: NP^−^/TUNEL^+^ cells.

Apoptosis is an innate cellular defense mechanism that has a critical role in preventing the growth of intracellular microbes, commonly found in viral and bacterial infections [28]. To evaluate apoptosis-mediated antiviral action by the extract, the cleavage ends of DNA fragments in the infected cells with A/PR8 strain were detected by TUNEL assay (Figure 4). As a result, the post-treatment of M20A3S10 extract successfully protected the host cell by activating the cellular apoptotic pathway, compared to the A/PR8-inoculated, vehicle-treated group. Moreover, the post-treatment remarkably decreased viral replication by 97% (from 23.742% of IAV-positive cells in vehicle-treated cells to 0.810% in the extract-treated cells). This data indicated that apoptosis-mediated innate immunity is a critical antiviral mechanism for the IAV infection.

### 3.5. Apoptosis-Mediated Antiviral Response Through M20A3S10 Extract Antiviral Activity of the M20A3S10 Extract Against ZIKV and DENV2

As enveloped RNA viruses, flaviviruses such as ZIKV and DENV share common replication mechanisms with influenza viruses, including viral attachment, entry, and membrane fusion processes [7]. For antiviral evaluation, Vero E6 cells were employed, as they are highly permissive to flavivirus infection. The use of epithelial cell lines in this study was based on their relevance to the natural tropism and replication dynamics of each virus [29]. While influenza viruses primarily target the respiratory epithelium, ZIKV and DENV initiate infection in the mosquito midgut epithelium and are also known to infect epithelial and endothelial cells in mammalian hosts [30]. Therefore, virus-permissive epithelial cell lines were selected to appropriately model each virus’s infection process.

**Figure 5:**
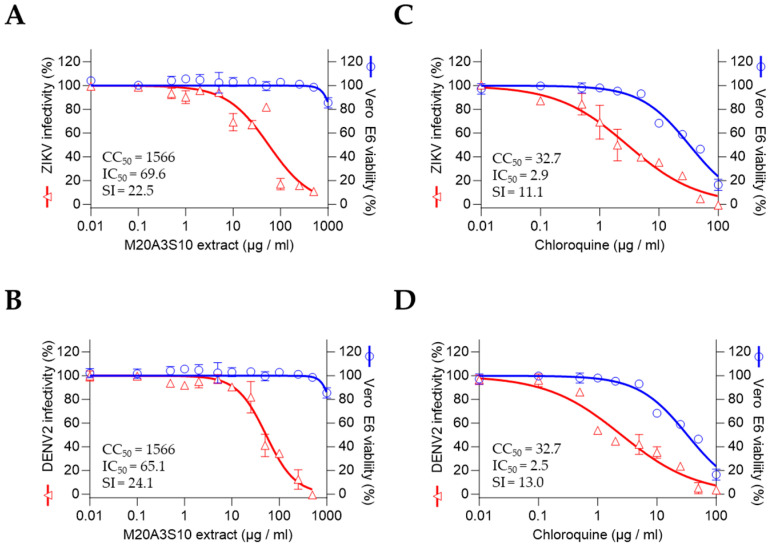
Antiviral activity against flaviviruses by full treatment of M20A3S10 extract. Antiviral actions were measured against ZIKV (**A**,**B**) and DENV2 (**C**,**D**) infections, treated with either the M20A3S10 extract or chloroquine.

To assess the therapeutic potential of the M20A3S10 extract against flaviviruses, antiviral screening was performed against ZIKV and DENV2. The results revealed a significant therapeutic index against ZIKV and DENV2 infections, with the extract exhibiting greater efficacy than chloroquine. The CC_50_ and IC_50_ values for the extract were found to be 1566 µg/mL and 696 µg/mL, respectively, with an SI of 22.5 for ZIKV and 1566 µg/mL and 65.1 µg/mL, with an SI of 24.1 for DENV2, with chloroquine exhibiting less therapeutic values than the extract for ZIKV (CC_50_ = 32.7, IC_50_ = 2.9, SI = 11.1), and 13 for DENV2 (CC_50_ = 32.7, IC_50_ = 2.5, SI = 13.0) (Figure 5).

**Figure 6:**
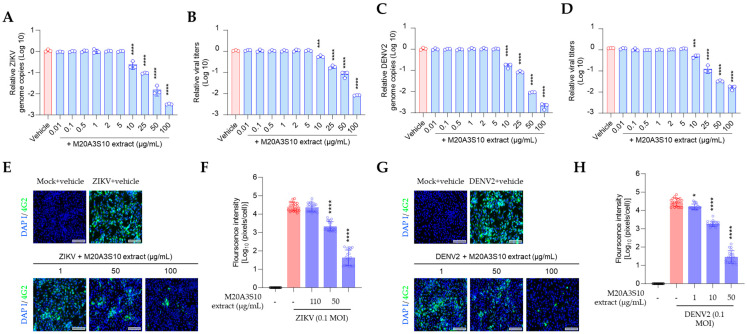
M20A3S10 extract efficiently suppressed flavivirus infections. (**A**,**B**) Genome replication (**A**) and progeny virus production (**B**) were inhibited by the extract in ZIKV infection. (**C**,**D**) Genome replication (**C**) and progeny virus production (**D**) were inhibited by the extract in DENV2 infection. (**E**,**F**) The extract inhibited the viral protein synthesis (**E**) in a dose-dependent manner, which was quantified by calculating the positive signal (**F**) in ZIKV infection. (**G**,**H**) The extract inhibited the viral protein synthesis (**G**) in a dose-dependent manner, which was quantified by calculating the positive signal (**H**) in DENV2 infection. *, *p* < 0.05; ***, *p* < 0.001; ****, *p* < 0.0001.

Pre- or co-treatment of the extract did not exhibit sufficient inhibition of those viruses. In contrast, post-treatment efficiently blocked the propagation of ZIKV and DENV2 in vitro (Figure 6). Dose-dependent inhibition was demonstrated in viral genome replication, progeny production, and protein synthesis. These findings suggest that the *Parerythrobacter* extract could effectively control IAV, ZIKA, and DENV2 infections.

### 3.6. Apoptosis-Mediated Antiviral Response in ZIKV and DENV2 Infections

**Figure 7:**
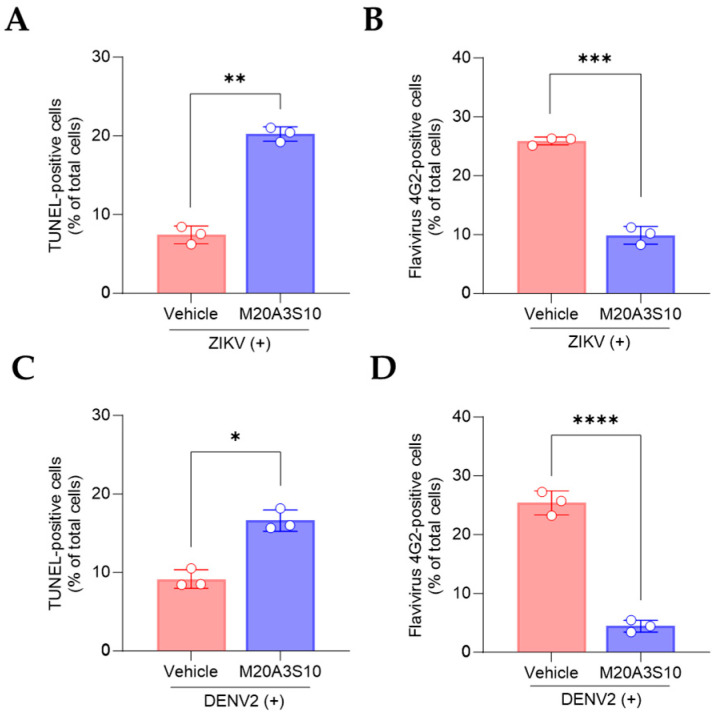
Evaluation of apoptotic response by M20A3S10 extract using flow cytometry. Apoptosis was measured by TUNEL assay and virus protein by an antibody against flavivirus group envelope protein, respectively. (**A**–**D**) Summary of flow cytometry data. Quantification of TUNEL (**A**,**C**) and virus-positive cells (**B**,**D**) treated with the vehicle or M20A3S10 extract. All data in the graphs are presented as the arithmetic mean ± S.D. from three independent experiments. *, *p* < 0.05; **, *p* < 0.01; ***, *p* < 0.001; ****, *p* < 0.0001.

Regarding the common replication mechanism, IAV, ZIKV, and DENV viruses prefer necroptosis over apoptosis to produce more progeny, severely damaging respiratory organs, neurons, and immune cells. Therefore, switching from necroptosis to apoptosis could be promising antiviral targets for broad infections. The post-treatment of the M20A3S10 extract increased apoptotic reactions and reduced viral replication (Figure 7), suggesting that apoptosis is an antiviral process limiting the replication of influenza and flavivirus.

### 3.7. A Broad-Spectrum Antiviral Activity Against Multiple Influenza Viruses

**Figure 8:**
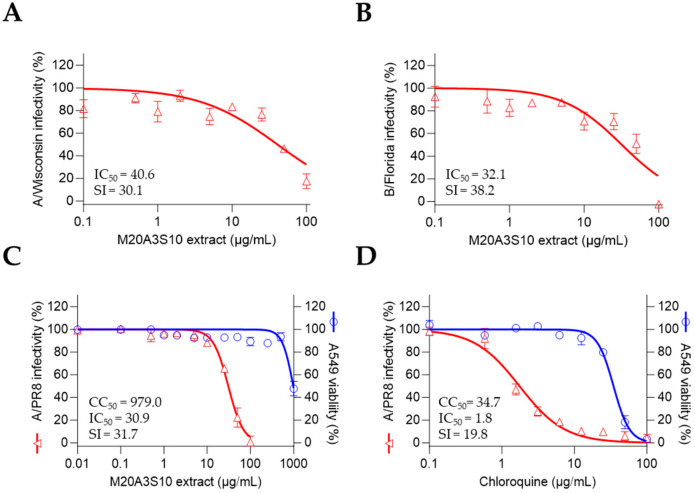
The post-treatment of the M20A3S10 extract shows a broad spectrum of antiviral potential against multiple influenza viruses. (**A**,**B**) Antiviral actions were measured against the A/Wisconsin strain (**A**) and the B/Florida strain (**B**). (**C**,**D**) Antiviral responses in A549 cells by the post-treatment of M20A3S10 extract (**C**) and chloroquine (**D**).

To further evaluate the breadth of antiviral activity, we tested the M20A3S10 extract against multiple influenza virus strains beyond A/PR8 (Figure 8). In addition, A549 cells—human alveolar epithelial cells—were used to assess the therapeutic applicability of the extract in a human cellular context.

As expected, the post-treatment of M20A3S10 extract dramatically exerted antiviral efficacies against influenza virus A/H3N2 (CC_50_ = 1223 µg/mL, IC_50_ = 40.6 µg/mL, SI = 30.1) and B/Florida (CC_50_ = 1223 µg/mL, IC_50_ = 32.1 µg/mL, SI = 38.2). Moreover, the extract more greatly protected the A549 cells from influenza virus A/PR8 (CC_50_ = 979 µg/mL, IC_50_ = 30.9 µg/mL, SI = 31.7) than chloroquine. These data suggest that M20A3S10 extract has in vitro broad-spectrum antiviral potential against multiple influenza viruses, which can be applied to various cell lines originating from different organs. These results indicate that the *Parerythrobacter* sp. M20A3S10 extract possesses broad-spectrum antiviral activity against multiple influenza virus subtypes in vitro, and its efficacy extends across epithelial cell lines of both animal and human origin.

## 4. Discussion

The ongoing threat of zoonotic RNA viruses such as IAV, ZIKV, and DENV highlights the urgent need for novel antiviral agents with broad-spectrum efficacy. These viruses exhibit high mutation rates, broad host ranges, and complex interactions with host immune systems, contributing to repeated outbreaks and significant veterinary and public health burdens. In this study, we evaluated the antiviral potential of a marine bacterial extract derived from *Parerythrobacter* sp. M20A3S10, isolated from coastal seawater. The extract exhibited notable in vitro antiviral activity against a diverse group of enveloped RNA viruses of veterinary importance, including IAV (H1N1, H3N2), IBV, ZIKV, and DENV2, suggesting a broad-spectrum mechanism of action.

From a veterinary immunology and pathogenesis perspective, a key finding was the extract’s capacity to inhibit viral replication by promoting apoptosis-mediated antiviral responses. This is particularly significant in the context of host–pathogen interactions: RNA viruses often hijack host cell death pathways to facilitate replication, shifting host cells toward necroptosis, which supports progeny production and inflammation [7,8]. Our data support the hypothesis that modulating this balance toward apoptosis—a more controlled form of cell death—can restrict viral replication and enhance phagocytic clearance of infected cells [6]. This insight provides a valuable immunological target for therapeutic intervention, especially for veterinary viruses that compromise immune organs and epithelial barriers.

Notably, post-treatment with the M20A3S10 extract was effective against all tested viruses, whereas pre- and co-treatment failed to prevent infection. Antiviral mechanisms associated with pre-treatment typically involve direct viral inactivation or interference with viral attachment to host cells. In contrast, co-treatment generally targets early stages of infection such as viral entry, membrane fusion, or endocytosis. However, neither pre- nor co-treatment suppressed viral replication, while post-treatment significantly reduced infection. These findings suggest that the antiviral effects of the extract are predominantly exerted at post-entry stages, likely during viral genome replication, viral protein synthesis, or interactions with host immune surveillance mechanisms.

This temporal pattern aligns with the activation of host apoptotic responses and implies that the extract’s active components may function by sensitizing infected cells to apoptosis or enhancing caspase-dependent cell death pathways [17]. Such a mechanism is particularly relevant, as apoptosis-mediated immune clearance represents a crucial antiviral defense in both human and veterinary hosts and serves as a promising therapeutic target in veterinary virology [31].

The extract’s efficacy was consistent across multiple influenza strains and cell types, including human and canine epithelial cells, which are representative of major target tissues in both human and veterinary infections. This cross-strain and cross-species efficacy supports the potential of this marine-derived compound as a versatile antiviral agent. Furthermore, its superior selectivity index compared to chloroquine indicates a more favorable therapeutic window, encouraging further pharmacological development.

To date, no studies have reported the antiviral activity of *Parerythrobacter* spp., underscoring the novelty of this study in exploring its potential as an antiviral agent. Although the specific active compound responsible for the observed effect remains unidentified, genomic analysis of *Parerythrobacter aurantius* has revealed the presence of a complete carotenoid biosynthesis gene cluster [32]. This finding supports the likelihood that *Parerythrobacter* spp. can produce bioactive pigments—such as carotenoids—with multifunctional properties, including potential antiviral and antioxidant effects.

In the context of epidemiology, the rising incidence of zoonotic and vector-borne viral diseases is exacerbated by climate change, wildlife–livestock–human interface expansion, and globalized movement of animals and goods. Natural compounds from marine microbes represent an underexploited resource for pandemic preparedness. Although *Parerythrobacter* is known to produce carotenoids with reported antiviral, anti-inflammatory, and antioxidative properties [11,33], the contribution of these compounds to the observed antiviral effects in this study remains hypothetical. Currently, there is no direct evidence linking the carotenoids present in the extract to its antiviral mechanism. Future studies should include pure compound isolation, validation, and gene knockout assays to confirm the active constituents. As the active components remain uncharacterized, it is also possible that other metabolites may contribute to the antiviral activity, either through direct inhibition of viral replication or by modulating the host immune environment. These findings highlight the need for further mechanistic investigations to elucidate both the molecular targets and the bioactive constituents involved.

Although the precise molecular constituents responsible for the observed effects remain to be identified, the data suggest that components of the extract—potentially carotenoids—interfere with late-stage viral replication and promote host-driven clearance mechanisms. Further metabolomic characterization (e.g., via LC-MS/MS) and in vivo efficacy testing in relevant animal models are warranted. Such studies could determine pharmacokinetics, bioavailability, and host-specific responses, which are essential for translational application in veterinary medicine. These experiments will begin using the A/PR8 (H1N1) strain, given its well-established utility in both in vitro and in vivo models [8]. In the future, additional studies leveraging the broad replication capacity of this virus may provide further insights into the antiviral potential of the extract.

Marine bacteria were chosen for this investigation due to their rich metabolic diversity, rapid growth in low-cost media, and proven capacity to produce antiviral pigments such as carotenoids [13,14]. While specific compounds were not isolated in this study, we aimed to showcase the scalability and feasibility of this resource. Notably, this study integrated a single screening platform to evaluate antiviral activity against IAV, IBV, ZIKV, and DENV2—viruses of both veterinary and zoonotic importance. Furthermore, flow cytometry-based dual quantification of viral protein and host apoptosis provided a mechanistic dimension rarely explored in previous studies.

In conclusion, the findings underscore the value of apoptosis modulation as an antiviral strategy and identify marine carotenoid-producing bacteria as promising candidates for the development of next-generation antivirals. Given the cross-species and broad-spectrum efficacy observed, this approach holds potential not only for companion animals and livestock but also for wildlife disease management and zoonotic outbreak containment.

## 5. Conclusions

In this study, we identified a marine bacterial extract from *Parerythrobacter* sp. M20A3S10 that exhibits broad-spectrum in vitro antiviral activity against several enveloped RNA viruses of veterinary and zoonotic concern, including IAV, IBV, ZIKV, and DENV. The extract was most effective when administered post-infection, suggesting that its antiviral action may involve interference with viral replication or modulation of host cell responses. The ability to inhibit viral proliferation in both human and canine epithelial cells underscores its potential relevance for veterinary antiviral development.

Although the precise bioactive constituents remain unidentified, bacteria of the *Erythrobacteraceae* family are known to produce compounds such as carotenoids with reported antiviral properties. While the presence of such metabolites in the tested extract has not yet been confirmed, their potential contribution warrants further investigation. These findings support the continued exploration of marine microbial resources as candidates for novel antiviral strategies, and future studies should focus on compound isolation, mechanistic analysis, and in vivo validation in relevant animal models.

## Data Availability

The data supporting the conclusions of this article are included within the article. Raw data are available from the corresponding author upon reasonable request.

## Supplementary Materials

The following supporting information can be downloaded at: https://www.mdpi.com/article/10.3390/ani15142125/s1, Figure S1: Antiviral effect of M20A3S10 extract by pre-treatment (A–C) and co-treatment (D–F) against A/PR8 strain. (A) Schematic diagram of virus inoculation and extract treatment. (B) IC_50_ and SI of M20A3S10 extract measured by CPE-inhibition assay. (C) Viral genome copies detected by RT-qPCR. (D) Schematic diagram of virus inoculation and extract treatment. (E) IC_50_ and SI of M20A3S10 extract measured by CPE-inhibition assay. (F) Viral genome copies detected by RT-qPCR. All data in the graphs are presented as arithmetic means ± S.D. from three independent experiments; Figure S2: Virus attachment (A) and penetration assay (B). (A) Viral genome copies by pre-treatment of the extract before virus binding. (B) Viral genome copies by post-treatment after virus binding. All data in the graphs are presented as arithmetic means ± S.D. from three independent experiments. HA, haemagglutinin. *, *p* < 0.05; **, *p* < 0.01; ****, *p* < 0.0001.
