# Supplementary material for: Therapeutic Use of Parerythrobacter sp. M20A3S10, a Marine Bacterium, Targeting Influenza Viruses and Flaviviruses
Source: Animals (Basel). 2025 Jul 18;15(14):2125. doi: 10.3390/ani15142125 (PMC12291660; doi:10.3390/ani15142125)
Supplement: Supplementary file 1 [file animals-15-02125-s001.zip › animals-3725937-supplementary.pdf]

Supplementary Materials

Supplementary Figures

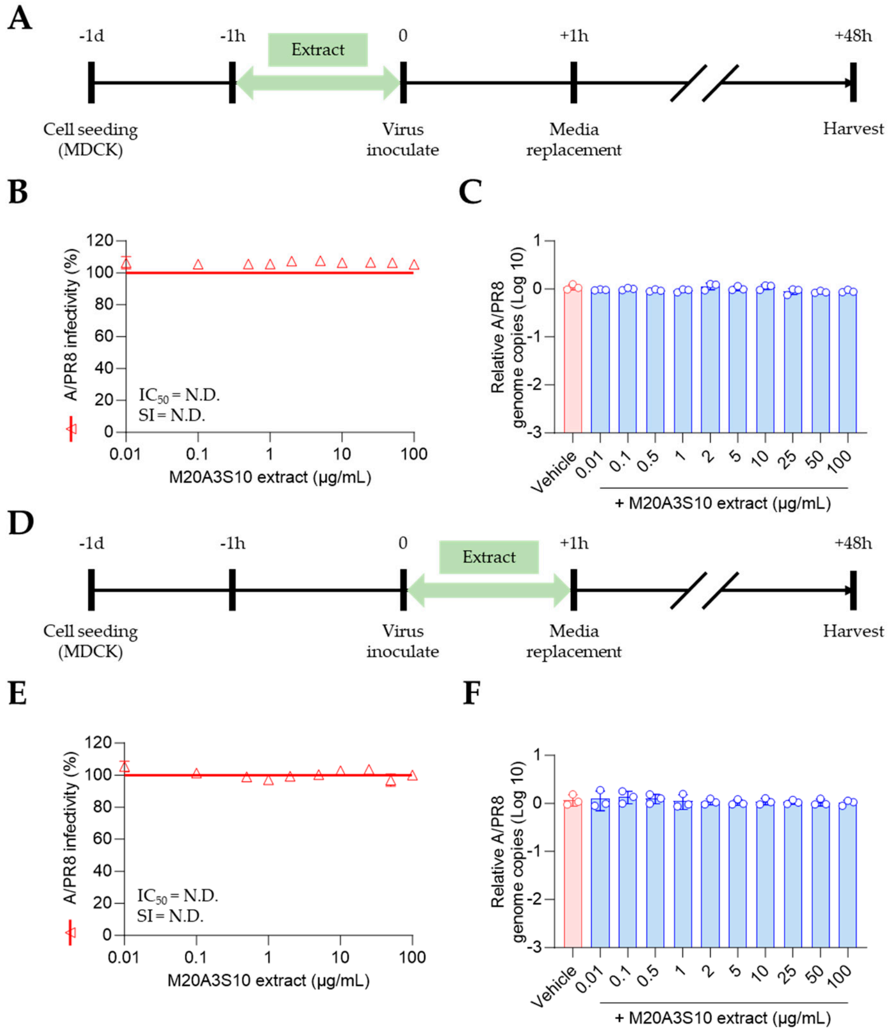

Supplementary Figure S1. Antiviral effect of M20A3S10 extract by pre-treatment (**A**-**C**) and co-treatment (**D-F**) against A/PR8 strain. (**A**) Schematic diagram of virus inoculation and extract treatment. (**B**) IC50 and SI of M20A3S10 extract measured by CPE-inhibition assay. (**C**) Viral genome copies detected by RT-qPCR. (**D**) Schematic diagram of virus inoculation and extract treatment. (**E**) IC50 and SI of M20A3S10 extract measured by CPE-inhibition assay. (**F**) Viral genome copies detected by RT-qPCR. All data in the graphs are presented as arithmetic means ± S.D. from 3 independent experiments.

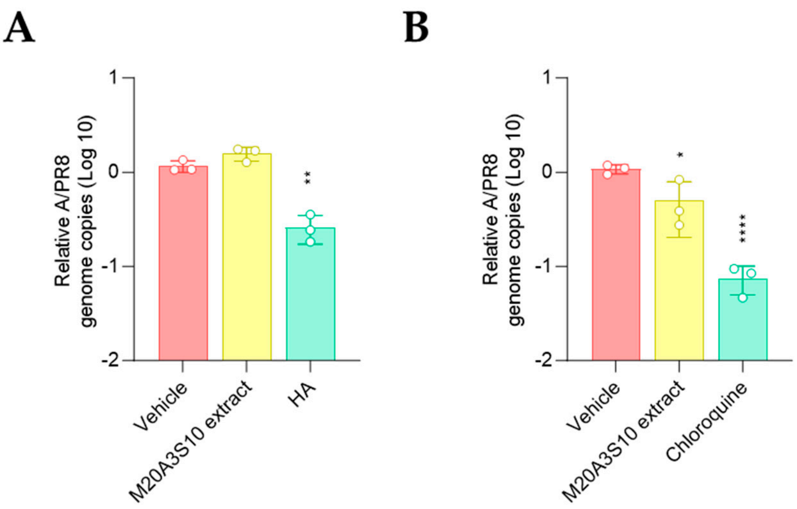

Supplementary Figure S2. Virus attachment (**A**) and penetration assay (**B**). (**A**) Viral genome copies by pre-treatment of the extract before virus binding. (**B**) Viral genome copies by post-treatment after virus binding. All data in the graphs are presented as arithmetic means ± S.D. from 3 independent experiments. HA, haemagglutinin. *, P<0.05; **, P<0.01; ****, P<0.0001.
